# Rotary Nanomotors in the Rear View Mirror

**DOI:** 10.3389/fmicb.2022.873573

**Published:** 2022-04-29

**Authors:** Michael D. Manson

**Affiliations:** Texas A&M University, College Station, TX, United States

**Keywords:** flagellum, rotation, motor, ion motive force, Howard Berg

## Abstract

Rotation is part of our everyday lives. For most of human history, rotation was considered a uniquely human invention, something beyond the anatomical capabilities of organisms. In 1973, Howard Berg made the audacious proposal that the common gut bacterium *Escherichia coli* swims by rotating helical flagellar filaments. In 1987, Paul Boyer suggested that the FoF1 ATP synthase of *E. coli* is also a rotary device. Now we know that rotating nanomachines evolved independently at least three times. They power a wide variety of cellular processes. Here, the study of flagellar rotation in *E. coli* is briefly summarized. In 2020, the Cryo-EM structure of the MotAB stator element of the bacterial flagellum was described. The structure strongly suggests that the MotAB stator rotates to drive flagellar rotation. Similar motors are coupled to other diverse processes. The following articles in this issue review the current knowledge and speculation about rotating biological nanomachines.

## Introduction

This article is intended as an introduction to an issue devoted to biological rotary nanomotors. It provides a historical perspective on the study of rotation of the bacterial flagellum, the first—and for more than 20 years the only—known biological rotary motor. It has recently become clear that rotary motors on a molecular scale are everywhere in nature. It is this realization that inspired the review and perspective articles that are compiled in this issue.

Rotation has some marvelous properties as a form of motion. It can be continuous in one direction as long as a constant force is applied to generate the torque required to balance the resistance. It is reversible, switching from clockwise to counterclockwise and back again (What did they call the two directions of rotation before we had clocks? Perhaps right-handed and left-handed screws). Rotation can be driven from torque applied by a central hub or axle, or it can be driven by an external current that supplies the energy for rotation about a hub or axle. It can generate or be driven by flows of wind, water, or electrical current. It is the ideal motion for conversion of potential energy into kinetic energy, and vice versa.

Rotation has been ubiquitous in our lives since the invention of the wheel, first as an aid to potters and only later as a convenient way to move heavy loads. Until 1973, we can be forgiven for thinking rotation was a uniquely human invention among living organisms. Examples of what superficially looked like rotation were illusions: the helical beat of the long, propulsive cilia of some protozoa; the seemingly circular beat of multiple, much-shorter cilia around the oral opening of a rotifer; the remarkably quick swiveling of an owl’s head through nearly 360°.

Everyone agreed there was a good reason that there was no rotation in living organisms. How would connections be made across a rotating joint? Blood vessels, nerves, connective tissue would all be twisted into an awful snarl, and any communication across the joint would be interrupted. Even at the microscopic level, filaments made of actin, myosin, tubulin, or any other polymerizing protein would become entangled. Another problem was how lubrication would be provided for parts rotating relative to one another.

The problem was that people were thinking too big. There is no need for a filamentous connection between the parts of a molecular motor. There is also no need for a lubricant.

The curtain that blocked our view of rotation in living organisms was raised in 1973. In a paper appearing in *Nature*, Howard Berg and Robert Anderson presented the idea that flagella, thin helical filaments used for motility by bacteria, propel the cells by rotating (Berg and Anderson, 1973). They reasoned from available information. The paper features one hand-drawn figure, thereby extending a precedent set by Watson and Crick 20 years earlier (Watson and Crick, 1953).

Berg and Anderson had two irrefutable pieces of evidence for rotation. A swimming *Escherichia coli* cell is pushed by a bundle of 4–6 flagellar filaments. The first evidence for rotation was provided by the observation that when divalent antibody prepared against flagellar filaments was added to swimming cells, the cells immediately stopped swimming. The interpretation was that their flagellar filaments became crosslinked and could no longer slide past one another as they must in a bundle of rotating filaments. When monovalent antibody raised against the filament was added, motility was retained. The interpretation was that the filaments became thicker when they bound the monovalent antibody but could still slide past one another as they rotated in a bundle.

**FIGURE 1:**
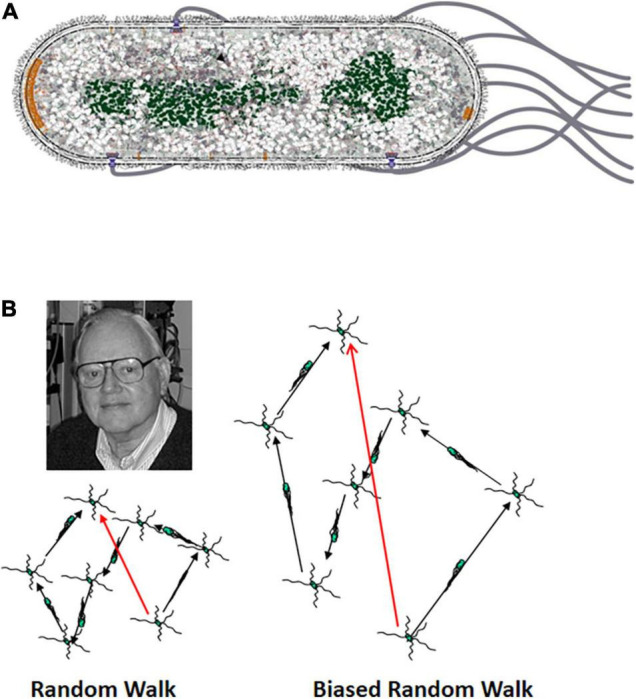
**(A)** Diagram of an *E. coli* cell. *E. coli* is a rod-shaped, gram-negative bacterium. It has an inner and outer membrane with a thin but relatively rigid peptidoglycan (PG) cell wall in the periplasmic space between the two membranes. The four to six flagella arise at random points along lateral surface of the cell cylinder. The proximal hook serves as a universal joint to allow rotation of the flagellum perpendicular to the cell envelope to bend 90° so that the left-handed helical flagellar filaments can come together to form a bundle that pushes the cell. The nucleoid is shown in green, and the chemoreceptor patches at the cell poles are shown in orange. The patch at the old pole is larger. In this image it is at the leading end of the cell, although the old pole can be either at the leading or trailing end (Berg and Turner, 1995). **(B)** The three-dimensional random walk. The image at the left shows a two-dimensional projection of a cell that is running and tumbling in three dimensions. The image to the right shows a cell that is swimming in a gradient of an attractant chemical with a higher concentration at the top. The runs in the up-gradient direction are longer than in the image to the left, whereas the runs in the down-gradient direction are of the same length. The shorter runs in the absence of an attractant gradient provide space for Howard Berg to look on approvingly from the upper left.

The second piece of evidence was again provided by divalent antibody, but in a very different way. An extracellular structure called the proximal hook is found at the base of the filament. This hook acts as a universal joint to allow rotation perpendicular to the lateral surface of the cell to bend 90° to allow the filament to rotate parallel to the long axis of the cell (Figure 1A). Polyhook mutants make extra-long hooks and no filament. These mutants do not swim. When divalent antibody raised against the polyhooks was added to a dense suspension of polyhook mutant cells, it was seen that pairs of adjacent cells became attached to one another. These cells began to spin in opposite directions, as they would if they were now joined by two crosslinked polyhooks, each rotated from its base.

This pioneering and paradigm-breaking work was quickly confirmed by experiments from the laboratories of Mel Simon and Julius Adler. Their papers were published back-to-back in *Nature* just 6 months after the Berg and Anderson paper appeared. Both took advantage of the fact that, if the stub of a flagellar filament or the proximal hook is attached to a surface by antibody, rotation of what soon came to be called the motor at its base caused the “tethered” cell body to spin. The Simon lab found that the cells rotated both clockwise (CW) and counterclockwise (CCW). Because the cell bodies must rotate in the opposite direction as the flagellum, this demonstrated directly that flagella rotate both CCW and CW (Silverman and Simon, 1974). The Adler lab showed that when tethered cells are exposed to chemicals that they sense as attractants or repellents, they respond by changing their pattern of rotation (Larsen et al., 1974b). Attractants cause the cells to rotate exclusively CW for a time because they cause the flagellum to rotate CCW. Repellents cause the cell bodies to spin exclusively CCW, although for a shorter time, because they cause the flagellum to rotate CW. This switching in the direction of flagellar rotation is the basis for chemotaxis (see below).

These discoveries came just at the time that the behavior of swimming cells was being recorded automatically by the tracking microscope invented by Howard Berg (1971). The tracking microscope had been used to show that *E. coli* cells swim in a behavior first called running and twiddling (Figure 1B), with twiddles later being called tumbles (Berg and Brown, 1972). The cells swim on a path (the run) that is as straight as can be given the rotational diffusion of an object as small as an *E. coli* cell. They then go through a brief period of chaotic motion (the twiddle or tumble) that reorients them before the onset of the next run. The average change in heading is about 68°, slightly biased in the direction the cell had been heading. Both runs and tumbles show an exponential length distribution, with the mean run lasting about 1 s and the mean tumble about 0.1 s. This behavior was aptly described as a 3-dimensional random walk.

The runs became longer when the cells swam up a concentration gradient of a chemical that was an attractant (Mesibov and Adler, 1972; Adler et al., 1973) or down a concentration gradient of a chemical that was a repellent (Tso and Adler, 1974). The data from the Adler lab with tethered cells made it clear that CCW flagellar rotation (viewed looking down the axis of the flagellar bundle toward the cell) propels runs and implies that CW flagellar rotation leads to tumbles. Cells swimming down an attractant gradient or up a repellent gradient do not change the length of their runs. This led Berg to conclude that *E. coli* is an optimist, happy when things are getting better and soldiering on unresponsively when things are getting worse. This differed from the classical idea that bacteria navigate by responding to negative stimuli with an avoidance response, the Schreckbewegung (shock reaction) described by Engelmann (1883).

The next major advance was the demonstration that flagellar rotation is powered by the ion motive force across the membrane. The Adler lab had shown as early as 1974 that an intermediate in oxidative phosphorylation, not ATP, was the driving force for flagellar rotation (Larsen et al., 1974a). In 1977, motility was induced in a suspension of non-motile de-energized cells of *Streptococcus* strain V4051 with a valinomycin-induced potassium diffusion potential or a transmembrane pH gradient (Manson et al., 1977). Thus, either the electrical or chemical component of the proton motive force (pmf) can drive flagellar rotation. Further confirmation of the role of the pmf in bacterial motility was provided by the Skulachev group (Glagolev and Skulachev, 1978).

Using de-energized tethered cells of the motile *Streptococcus*, it was demonstrated that the rotational speed of the flagellar motor operating at high load is directly proportional to either the electrical or chemical component of the pmf (Manson et al., 1980). In these experiments, the highest value of the pmf was −80 mV. Rotation was induced even when the sign of the pmf was reversed so that hydrogen ions flowed out of, rather than into, the cells.

A similar result was obtained using voltage-clamp experiments with giant *E. coli* cells (Fung and Berg, 1995). In this study, the linear dependence on pmf was extended to −150 mV, nearly the −170 mV pmf maintained by actively metabolizing *E. coli* cells. It was also shown that an interior positive pmf drove CW rotation of a flagellum that rotated CCW in response to an inwardly directed proton flow. This result confirmed a conclusion reached earlier with tethered cells of a *Streptococcus* mutant whose flagella were locked in CCW rotation. Their flagella spun CW when an inside-positive potassium diffusion potential was induced with valinomycin in the presence of a high external KCl concentration (Berg et al., 1982).

The motile *Streptococcus* had one more contribution to make to our understanding of flagellar rotation. It was used to estimate the proton flux through the flagellar motor (Meister et al., 1987). The data suggested that about 1,200 protons were required per rotation of the flagellum. This result led to the conclusion that when the motor operates at high load, as in a tethered cell, it works with close to 100% efficiency. For rapidly spinning flagella, the efficiency is much lower, probably “due to dissipation by processes internal to the motor.”

**FIGURE 2:**
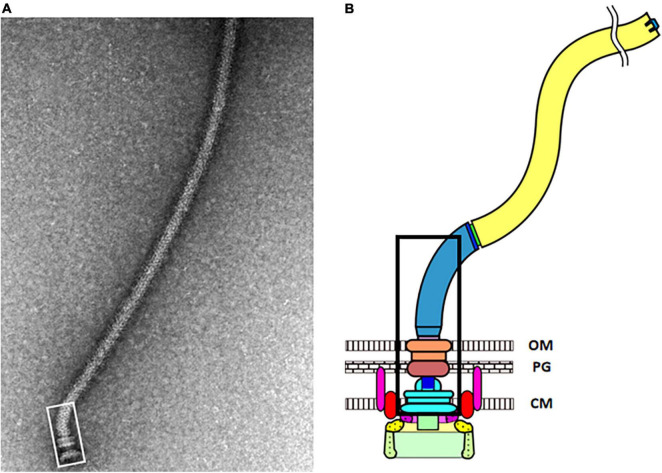
**(A)** Electron micrograph of an isolated flagellum. The vast majority of the photo shows the long flagellar filament. The hook-basal body complex is outlined by the white rectangle at the lower left. It contains the MS-ring, the rod, the P-ring, the L-ring, and the proximal hook. The C-ring, the export apparatus, and the MotAB stators did not co-isolate with the hook-basal body complex. **(B)** Cartoon image of an *E. coli* flagellum. The portion of the hook-basal body outlined by the white rectangle in panel **(A)** is outlined by the black rectangle. Two MotAB stator units are shown, with MotA in red and MotB in pink. Below the MS-ring (light blue) is the export apparatus show in purple and as a green rectangle. FliG (yellow) is at the top of the C-ring, where it can interact with MotA. FliM and FliN (light green) are below FliG (Images kindly provided by Tohru Minamino, Osaka University).

The bacterial flagellum is a complex machine (Figure 2). In *E. coli*, the products of about 40 genes (Table 1) are required to construct a fully functional flagellum. In other species, particularly those with very rapidly rotating flagella (e.g., *Vibrio* species) or internal periplasmic flagella (spirochetes), other proteins are required, but the basic set of parts remains the same. Most of the gene products are designated as Flg, Flh, or Fli, depending on where on the chromosome the encoding genes are located. Four of these proteins are transcriptional regulators. Others are required only transiently during the assembly of the flagellum. Two of them, MotA and MotB, are not required to build a flagellum but are required for it to rotate. They are of special interest because they comprise a common element, presumably evolved from a common ancestral gene, that is found in several rotary motors discussed in this issue that are coupled to processes other than flagellar rotation.

**TABLE 1:** Proteins involved in the regulation, assembly, and structure of the *E. coli* flagellum.

Protein	Function	Component of mature flagellum	Operon	Map location (min)
FlgA	P-ring assembly	No	*flgA*	24
FlgB	Proximal rod	Yes	*flgB-J*	24
FlgC	Proximal rod	Yes	*flgB-J*	24
FlgD	Hook cap	No	*flgB-J*	24
FlgE	Hook protein	Yes	*flgB-J*	24
FlgF	Proximal rod	Yes	*flgB-J*	24
FlgG	Distal rod	Yes	*flgB-J*	24
FlgH	L-ring	Yes	*flgB-J*	24
FlgI	P-ring	Yes	*flgB-J*	24
FlgJ	Muramidase	No	*flgB-J*	24
FlgK	Hook-filament junction (at hook)	Yes	*flgKL*	25
FlgL	Hook-filament junction (at filament)	Yes	*flgKL*	25
FlgM	Inhibitor of σ*^F^*	No	*flgMN*	24
FlgN	Chaperone for FlgK and FlgL	No	*flgMN*	24
FlhA	Protein export	Yes	*flhBAE*	42
FlhB	Protein export specificity	Yes	*flhBAE*	42
FlhC	Master transcriptional regulator	No	*flhDC*	42
FlhD	Master transcriptional regulator	No	*flhDC*	43
FlhE	Regulation of protein exporter	Yes	*flhBAE*	43
FliA	Sigma factor for late flagellar genes (σ*^F)^*	No	*fliA*	43
FliC	Flagellin (polymerizes to form filament)	Yes	*fliC*	43
FliD	Filament cap (needed for FliC polymerization)	Yes	*fliDST*	43
FliE	MS-ring rod junction	Yes	*fliE-K*	43
FliF	MS-ring	Yes	*fliE-K*	43
FliG	C-ring, interacts with MotA	Yes	*fliE-K*	43
FliH	Protein export	Yes	*fliE-K*	43
FliI	Protein export (ATPase)	Yes	*fliE-K*	43
FliJ	Export chaperone	Yes	*fliE-K*	43
FliK	Hook-length control	No	*fliE-K*	43
FliL	Stability of flagellum	Yes	*fliL-R*	44
FliM	C-ring, switch component, binds CheY-P	Yes	*fliL-R*	44
FliN	C-ring, switch component	Yes	*fliL-R*	44
FliO	Scaffold for FliPQR assembly	No	*fliL-R*	44
FliP	Protein export	Yes	*fliL-R*	44
FliQ	Protein export	Yes	*fliL-R*	44
FliR	Protein export	Yes	*fliL-R*	44
FliS	FliC chaperone	No	*fliDST*	43
FliT	FliC chaperone	No	*fliDST*	43
MotA	Stator component, interacts with FliG	Yes	*motAB^a^*	43
MotB	Stator component, bind to PG cell wall	Yes	*motAB^a^*	43

*^a^The complete operon is motABcheAW.*

The first part of the flagellum to assemble is the membrane-inserted MS-ring. It is composed of 26 copies of the FliF protein. The rod, the proximal hook, and the filament extend outward from the MS-ring. The C-ring, made up of the FliG, FliM, and FliN proteins, assembles on the cytoplasmic face of the MS-ring. FliM and FliN interact with the signaling protein CheY, which is converted into its active phosphorylated form at the chemoreceptor patch, to switch the direction of flagellar rotation from CCW to CW. FliG, which is attached firmly to FliF in the MS-ring, interacts with MotA, which supplies the force needed for rotation of the C-ring.

MotAB constitute the ion channel and form the stator component of the flagellar motor. They function as stators because the C-terminal portion of the MotB protein binds tightly to the peptidoglycan (PG) cell wall. Stators can be added to induce the rotation of paralyzed flagellar basal bodies that are already assembled but not functioning (Silverman et al., 1976). When the flagellar motor operates at high load, as in a tethered cell, the addition of each stator causes an equal step increase in rotation speed (Block and Berg, 1984). The original estimate was that up to 8 MotAB units could be added to a motor (Blair and Berg, 1988), but later work raised that estimate to a maximum of at least 11 stator units per motor (Reid et al., 2006). The flagellar motor can operate at low load with a single stator unit, but additional stators are recruited when increased load on the motor requires the production of higher torque (Lele et al., 2013; Tipping et al., 2013).

Charged residues in a cytoplasmic loop of the MotA subunit of the stator make electrostatic contact with charged residues in the C-terminal “motility” domain of the FliG protein to apply torque to the C-ring (Zhou et al., 1998a). In *E. coli*, the association of stator units with the flagellum is dynamic. There is a rapid exchange between motor-associated stator units and a pool of dissociated stator elements freely diffusing in the membrane (Leake et al., 2006). The dwell time of a stator unit on a motor in that study was about 30 s.

When the stator units are in the membrane and not associated with a flagellar motor, a plug in the MotB subunit blocks the flow of protons (Hosking et al., 2006). A radical refolding of the periplasmic domain of MotB occurs when a stator unit associates with the C-ring (Kojima et al., 2009). This conformational change does two things; it unplugs the ion-conducting channel in the stator, and it extends the PG-binding domain so that it can bind to the PG cell wall. Thus, anchoring of the stator and its activation as an ion channel are coupled events.

The stoichiometry of MotA and MotB was thought to be 4:2 (Kojima and Blair, 2004). It is striking how long that misconception persisted and perhaps delayed the discovery that the stator rotates. An aspartate residue near the cytoplasmic end of the membrane-spanning N-terminal segment of MotB is critical either as a site of protonation (Zhou et al., 1998b) or of Na^+^-binding to the MotB homolog PotB in sodium-driven motors (Fukuoka et al., 2009). Transmembrane helices 3 and 4 of MotA and the single transmembrane helix of MotB form the ion-conducting channel (Braun et al., 2004). Most models for stator function envisioned the cytoplasmic loop of MotA swinging back and forth like the arm of a parent propelling children on a playground merry-go-round. That is how I described it in my bacterial physiology class. It remained a puzzle, however, why twice as many channel elements were present in MotA as in MotB. Were two of the MotA proteins just loafing some of the time?

The original estimate of A to B subunit stoichiometry in the stator was based on biochemical analysis of purified stator complexes. However, carefully those experiment were carried out, they cannot match the accuracy of actually imaging the stator complexes with cryo electron microscopy. Two groups accomplished that in 2020 (Deme et al., 2020; Santiveri et al., 2020). Both studies agreed that the stoichiometry is 5 MotA to 2 MotB subunits rather than 4:2. The new 5:2 ratio suggested that only one effective ion-conducting channel exists at a time and led to immediate speculation that what must be happening is that the MotA pentamer rotates around the MotB dimer in a temporal series of 10 steps of 36° in a full rotation.

**FIGURE 3:**
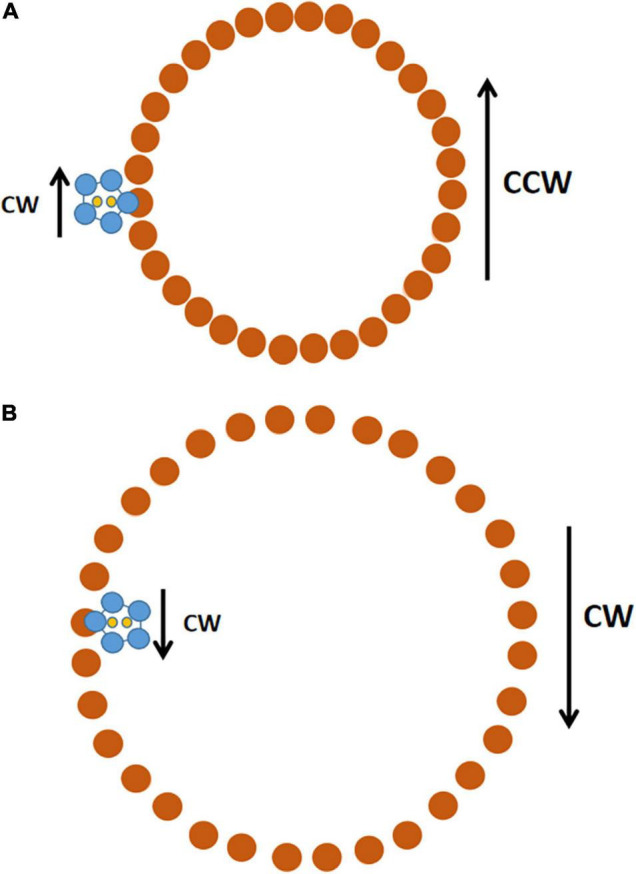
Schematic of how a CW-rotating stator can drive both CCW and CW rotation of the C-ring. The view is looking down from the cell membrane toward the top of the C-ring. **(A)** The C-ring is in its compact conformation. In the stator unit (MotA pentamer in blue, MotB dimer in yellow), the MotA pentamer rotates CW around the MotB dimer, which is fixed in place by its attachment to the PG cell wall. The inner edge of the MotA ring contacts FliG (brown) in the C-ring and drives its CCW rotation. There are 34 FliG subunits in the C-ring (Hu et al., 2022); the cartoon shows 32 because it was easier to get 32 evenly spaced. **(B)** The C-ring is in the expanded conformation it assumes after binding of CheY-P to FliMN. The stator unit remains fixed in the same position, but now, when the MotA pentamer rotates CW, its outer edge makes contact with FliG and drives rotation of the C-ring in the CW direction. Only one stator unit is shown, but there is room for up to 11 stators to associate with the C-ring.

The contacts between MotA and FliG would allow rotation of the stator to drive rotation of the C-ring, and thus of the entire flagellum (Hu et al., 2022). The stators presumably rotate only CW, but they can drive the rotation of the C-ring in either direction (Figure 3). If the C-ring is in a compact configuration, the CW-rotating stators would make the C-ring rotate CCW. If the C-ring is in an expanded configuration, the CW-rotating stators would make the C-ring rotate CW.

This was one of those amazing moments in science when one discovery solves multiple problems. The first is how a flow of ions through the stator into the cell translates into rotation of the C-ring. The second is how flagella can rotate bidirectionally when the ion flow is normally always from outside the cytoplasm to inside. The third is why a flagellum that normally rotates only CCW reverses to CW rotation when the proton motive force is inside positive; if protons flow outward through the stator, it should rotate CCW and drive CW rotation of the C-ring fixed in the compact conformation.

It was the discovery of the rotating stator that provided the impetus for compiling an issue devoted to rotary nanomachines. Various aspects of flagella are the topics of seven articles. Four articles consider systems that operate with homologs of MotAB that are coupled to functions other than flagellar motility: movement over surfaces by gliding bacteria, transport of large substrates across the outer membrane of gram-negative bacteria, and cell division. Two articles consider a structure that superficially looks like a flagellum but is functionally and evolutionarily completely distinct: the ATP-hydrolysis-driven archaellum of the Archaea. Finally, seven articles discuss the rotary FoF1 ATP synthase. Evidence to support the idea that this enzyme in *E. coli* is a rotary device was presented by the laboratory of Paul Boyer in 1987 (Kandpal and Boyer, 1987) and demonstrated to be a rotary device by direct observation in 1997 (Noji et al., 1997). The energy for all of these rotary machines other than the archaellum is provided by a transmembrane ion motive force that drives the coupling ion down its potential energy gradient into the cell. This issue celebrates both rotary nanomachines and the life of Howard Berg, who departed us on December 30, 2021. Enjoy.

## Author Contributions

MM conceived and wrote the article.

## Conflict of Interest

The author declares that the research was conducted in the absence of any commercial or financial relationships that could be construed as a potential conflict of interest.

## Publisher’s Note

All claims expressed in this article are solely those of the authors and do not necessarily represent those of their affiliated organizations, or those of the publisher, the editors and the reviewers. Any product that may be evaluated in this article, or claim that may be made by its manufacturer, is not guaranteed or endorsed by the publisher.
